# Reply to Heinschke, S.; Schneider, J.J. Comment on “Pashchanka, M. Conceptual Progress for Explaining and Predicting Self-Organization on Anodized Aluminum Surfaces. *Nanomaterials* 2021, *11*, 2271”

**DOI:** 10.3390/nano13212877

**Published:** 2023-10-30

**Authors:** Mikhail Pashchanka

**Affiliations:** MSE, Faculty 3mE, Delft University of Technology, Mekelweg 2, 2628 CD Delft, The Netherlands; mikhail.pashchanka@gmail.com

First of all, I would like to thank Silvio Heinschke and Jörg J. Schneider (hereinafter referred to as “the Readers”) for preparing their Comment [1]. It is immediately obvious that lots of thought and effort were put into creating that piece of writing.

Before going into the main points of my reply, I must emphasize that my approach to the consideration of all models in the review article under discussion was purely professional. While the Readers’ Comment contains such claims as “… *a critical analysis of the review reveals shortcomings in the scientific reasoning …*”, I would like to assure them that I relied solely on logical argumentation and generally accepted criteria of scientific quality. Although the Readers write that “*… MP’s arguments draw a distorted picture of our article*”, the consideration of their model was unbiased and based on common rules of science.

Concerning their remarks as to the quoting out of the “*broader context*” and “*deviating from the actual content*”: there is an established culture of scientific citation. An author does not have to cite bulky fragments of the original text completely. Appropriate usage of borrowed excerpts involves providing references for a reader to go directly to the sources (as was carried out in the review article in *Nanomaterials*). To illustrate the point, we may take the following sentence in the Readers’ Comment: “*On p. 17 of the review MP wrote “entropy deals specifically with irreversible, time-oriented processes”, which is to be doubted considering that equilibrium processes are also assigned to a specific entropy value*”. The original context can be reconstructed from the source: “*Entropy deals specifically with irreversible, time-oriented processes* (*irreversible processes produce entropy, i.e., the overall entropy change of the system and its surroundings will be positive and non-zero*)” [2]. Thus, a reader actually receives the essential information about the difference between irreversible and reversible processes—namely, that irreversible processes generate entropy, whereas reversible processes do not generate it (but they may transfer entropy from one part of the universe to another). I certainly did not see such criticism coming because this particular sentence deals with the relatively simple aspects that are tackled in classical textbooks for undergraduates. The second law of thermodynamics for reversible processes is “the law of entropy existence and conservation” (Δ*S* = *Q*/*T*), whereas for irreversible processes it is “the law of entropy existence and increase” (Δ*S* > *Q*/*T*).

It is worth mentioning here that the Readers also published a follow-up paper about their calculations in 2022 [3]. Although they were obviously aware of the critical remarks made in the review in *Nanomaterials* one year earlier, this review was never cited in their later article. If the Readers are confident in their findings, the reasons for such “cherry picking” and deliberate ignoring of the earlier works that pinpoint weaknesses in their considerations are not entirely clear.

Regarding the substantial part of the Readers’ article published in 2020, it reveals a lack of understanding of some fundamental principles despite excessive formalism [4]. Development of a mathematical model of a physical-chemical process should include problem statement, justification of a model, construction of a model, and, finally, the problem solving. The Readers’ paper creates the impression that they have concentrated their effort on the last step and on the overall “scientism” of their report, without paying due attention to completion of the previous steps and making sure that their model does not violate the laws of nature. It looks as if they were in a rush to borrow the semi-empirical formula from the electroconvective theory (reported in 2011) and to take into account the universal dependence of *P* on the concentration of charge carriers (that was experimentally investigated and published in 2016), to turn it into a new equation and to “intercept the palm of victory” [5,6,7].

I want to say I firmly support theoretical endeavors in this direction. Experimental verification has demonstrated more than once that the electroconvective theory yields testable predictions and is therefore safe to develop it further. Based on the results reported in 2016, the expression of *P* in terms of the ionic strength (or some similar element containing charges and concentrations of ions in solution) was a purely technical matter. I am actually not opposed to further development of this direction by any research group, and I also encouraged potentially interested researchers to pay attention to this theoretical problem in my review article. However, the number of incorrect assumptions made by the Readers in order to fit their merely mathematical results to the previously obtained experimental data is very large. Such mistaken assumptions are not confirmed either theoretically or experimentally. They are also not justified by our actual knowledge of the anodizing process, properties of electrolyte solutions, electrochemistry in general, as well as the first principles of thermodynamics. There is an odd situation when an incremental and “low risk” research work with plenty of mistakes is presented as having intellectual scope to complement or even radically change our understanding of the mechanisms involved in porous anodic alumina (PAA) self-organization. What is more, the Readers are now even expecting me to reconsider the well-established electroconvective theory after the publication of their inappropriate mathematical model based on incomplete or incorrect reasoning: “*MP doesn’t question his original assumptions in the light of our theory*”.

I give credit to the Readers for the processing and interpolation of a considerable amount of data previously obtained by other investigators. However, I have also analyzed their model in terms of physical and chemical laws and concepts and explained the multiple reasons why it is inconsistent [2]. Unfortunately, the Comment by the Readers indicates that critical views have not been taken into consideration for a radical rethinking of their approach. Under such circumstances, I deem it necessary to continue listing reasons why their model is invalid. Please, see the arguments below.

## 1. The Readers’ Model: A Perpetual Motion Machine of the Second Kind

This section analyzes the system modelled by the Readers and described on page 2 of the Comment. Briefly, the following conditions were specified:

1. The system is a portion of electrolyte solution confined between two electrodes and surrounded by the rest of the solution in the bath (I will use a metaphor: like a yolk in an egg);
2. The system is kept under isothermal conditions: “*the system is assumed to have a constant temperature* (*as well as the surrounding bath*)”;
3. There is no heat exchange between the system and its surroundings: “*… the heat exchange between bath and system is 0 … and the system is isolated against a heat exchange with the environment, which is the atmosphere around the bath …*”;
4. Since there is no heat transfer between the system and the rest part of the bath, there are also no associated changes in entropy (formulated by the Readers as $\frac{d_{en}S}{dt}=0$);
5. The bath (i.e., the “egg white”) is in contact not only with the atmosphere, but also with a cryostat “*which exchanges the heat from the solution, which is created during the process, with the environment.*” However, the system (the “egg yolk”) itself is located at some distance from a cryostat, and heat exchange between them (through the “egg yolk’s boundaries” and through the bulk of the “egg white”) is forbidden: Δ*Q* = *Q*_system_ − *Q*_bath_ = 0;
6. The Readers do not deny that a certain amount of heat is generated at the electrodes—the physical boundaries of their system, although it does not appear in their calculations. In their work, they wrote: “… *our model does not allow local heating of the electrode to be considered*” [4].

Let us now consider the system from the thermodynamic standpoint. It is separated from the surrounding bath by both the physical boundaries—the electrode surfaces—and the imaginary boundaries. Energy enters the system as electrical work (it is transformed into electrical current, formation of electrolysis products, self-organization, etc.), and heat generated at the electrodes. This heat *Q* absorbed by the system goes partly to increase internal energy Δ*U* and partly to do work *W* (the first law of thermodynamics):

*Q* = Δ*U* + *W* (1)

Given that the process is isothermal (see the second item on the list of conditions, *T*_system_ = const), there are no changes in the internal energy (Δ*U* = 0). The heat also cannot be transferred from the system to the bath because of the third item on the list of conditions (Δ*Q* = 0), and thus also cannot be transmitted through the bath towards the atmosphere or a cryostat (the fifth item on the list). Consequently, the received heat *Q* can leave the system only after its complete transformation into work *W* (from the first principle of thermodynamics: *T*_system_ = const, Δ*U* = 0, therefore *Q* = *W*). Such transformation is possible due to the hydrogen evolution reaction at the cathode:

2H^+^ + 2e^−^ → H_2_ (2)

The work performed by the system is the work of volume expansion (or so-called *pV*-work):

*W* = Δ*nRT* (3)

where Δ*n* is the amount of hydrogen gas that was released to the atmosphere.

Let us now proceed to the formulation of the second law of thermodynamics given by Wilhelm Ostwald: “*It is impossible to construct a perpetual motion machine of the second kind—a device that would perform work solely by absorbing energy as heat from a body. Some portion of the energy absorbed as heat from a source must always be rejected to a low temperature sink*”.

Thus, the Readers have constructed a “Perpetuum mobile” that absorbs heat generated by the electrodes and turns it into *pV*-work completely, without any possibility to give a part of *Q* to a low temperature sink (i.e., to a cryostat or the atmosphere, depending on what temperature of the bath is maintained: 0–5 °C or 35–60 °C) [8,9].

I have used Ostwald’s formulation here to emphasize the importance of the heat transfer through the imaginary boundaries of the system towards the cryostat, and the absurdity of the condition “Δ*Q* = 0”. Of course, the Readers’ model violates the second law written also in its more habitual form (compiled from the definitions given by Clausius, Thomson and Ostwald): “*No process is possible in which the sole result is the absorption of heat from a reservoir and its conversion into work*”.

It is also noteworthy that the maximum work is performed by a system in a reversible process. The less work is performed (i.e., the more energy is dissipated as heat), the more irreversible is a process. Entirely irreversible processes generate no work at all. The Readers are investigating an irreversible process. Consequently, the dissipation of heat from the system is an essential part of the process that cannot be safely ignored. I will give an experimental fact that could possibly help the Readers to rethink their model in the future and to reconsider the condition “Δ*Q* = 0”: when I performed hard anodizing experiments in oxalic or phosphoric acid electrolytes, the cooling capacity of a cryostat was insufficient, and the cell temperature rose rapidly. The temperature increase could be registered irrespective of the point of the thermometer immersion: it was higher in both the Readers’ system (the “egg yolk”) and in the surrounding bath (the “egg white”), which means that the heat exchange cannot be safely neglected.

It is also important that the heat generation at the anode will increase the entropy in the self-organizing system (making it closer to a “heat death” moment). Entropy is a function that cannot be restored to its initial value without the heat elimination, as formulated by Carathéodory principle.

Even without taking into account the heat generated at the electrodes, the rigidly set conditions of no heat exchange (*Q* = 0) and of a constant temperature (Δ*U* = 0 because no internal energy change occurs in an isothermal system) make the modelled system incapable of performing any useful work, including the work required for internal self-organization processes. The first law of thermodynamics *Q* = Δ*U* + *W* takes the form:

0 = 0 + *W* (4)

which makes the system “absolutely useless” from the thermodynamic standpoint.

I would like to conclude this section by reminding the Readers’ motivation for their research, which is written in the Abstract part of their paper: “*To the best of our knowledge, there is no model that establishes a theoretical connection between the long-range order of the PAOX system and its physico-chemical properties*” [4]. Thus, it is very strange that they started from removing such most important physico-chemical parameters from consideration, moving from accurate science to some sort of creationist thinking.

## 2. A Non-Existent Type of Thermally Isolated, Adiabatic Systems That Allow Exchange of Matter with the Environment

This section discusses the properties of the modelled system boundaries, as described by the Readers in the following fragment of their Comment: “*Additionally, the term adiabatic used by MP doesn’t apply to the experimental situation defined in our paper, because even in the case of the total isolation of the system against heat exchange, the definition* “*is held at constant temperature*” (*see our paper p. 11915*) *refers to the point, that the bath has to be kept at the considered temperature. In that way the generated heat is compensated. In reality, this is normally done by a cryostat which exchanges the heat from the solution, which is created during the process, with the environment*”;

Such a description is somewhat conflicting because “*the total isolation of the system against heat exchange*” co-exists with the heat exchange between the system and the environment mediated by a cryostat. Moreover, this description contains a free interpretation of strict thermodynamic definitions: if there is no heat flow between the system and the surroundings (Δ*Q* = 0), then the process in such a system is called “adiabatic”, and the boundary is called an “adiabatic shell”.

The system modelled by the Readers (the “egg yolk”) is separated from the rest of the solution in the bath (the “egg white”) by an imaginary boundary that does not pass heat, but is permeable for the molecules of water and the electrolyte ions: they can pass freely through the boundary because it does not physically exist. The particles from the system and from the bath can also chaotically collide at the boundary, but without energy exchange because of the constraint “Δ*Q* = 0” (this, in fact, contradicts the fundamental mechanical definition of heat).

Mass transfer between the system and the atmosphere is also allowed (the release of H_2_ gas from the space at the cathode). However, the heat exchange between the system and “*the atmosphere around the bath*” is also forbidden in the model.

There are three types of thermodynamic systems that are classified according to the types of allowed exchange with their surroundings: open (both matter and energy exchanges are allowed), closed (only energy), and isolated (nothing can be exchanged). Let us now consider the system modelled by the Readers:

- Matter exchange (water, electrolyte, hydrogen gas) between the system and the surrounding is allowed, therefore, the system is not closed or isolated.
- Energy can enter the system as both work (electrical) and heat (from electrode heating), but it can leave the system only as work of expansion (H_2_ release to the atmosphere), which is impossible according to the second law of thermodynamics. Thus, it is also not sustainable as an open system.

Thus, the Readers have modelled a system that does not exist in thermodynamic classification: it is thermally isolated and open at the same time.

As far as I understand, the wish of the Readers to artificially isolate the “system” from the surrounding objects could be in part motivated by the works in Synergetics by Hermann Haken. In a book section called “Master Equations and Limitations of Irreversible Thermodynamics” he mentioned that the approach used by the irreversible thermodynamics can be only applied if we assume no interaction between sub-systems of a system, or, using the terms of statistics, the correlation between them is negligible. However, such interactions are negligible on the macroscopic level not for every system. This is why Haken discussed the “Limitations” [10].

## 3. The Applicability of Ohm’s Law for the Calculation of the Specific Conductivity of Solution

I have carefully read the Readers’ reasoning regarding “*the mathematical body in the context of our theory*” and would say that this reasoning is often incomplete or incorrect, and many phenomena actually exist beyond the paradigm of such mathematical exercises detached from physical reality. In the model under discussion, this is particularly relevant for electrical properties of water solutions that are ionic conductors.

- For instance, the Readers write: “*As this is done on the basis of the assumption, that the process is in a stationary state with a constant current i(t_c_) (i.e., a constant amount of H^+^-ions reduced per time at the cathode), potential (potentiostatic process) and current are both constant and so is the resistivity, i.e., Ohm’s law is applicable in this case.*” The absurdity of this entire sentence becomes clear if we recall what an Ohmic conductor is and what a non-Ohmic conductor is. If the resistivity (and resistance) relates the applied voltage to the arising current linearly, i.e., it remains constant within certain ranges of Δ*U* and *I*, then Ohm’s law is met (Δ*U* = *IR* is a straight-line equation). If the resistivity changes within this range, and an *I* − Δ*U* curve is nonlinear, then Ohm’s law is not met.
  The Readers take only one single point on an *I* − Δ*U* curve where *I* = const = “*i*(*t_c_*)” and Δ*U* = const, and based on this single point conclude that Ohm’s law is met. Such incorrect arguments cast doubts on the credibility and the adequacy of the mathematical model as a whole;
- The Readers also write that “*i(t_c_) can just be replaced by an inverse exponential function*”. So, this means the end of applicability of Ohm’s law even in the eyes of the Readers? Resistivity is no more constant?

In the review paper, I have explained that Ohm’s law is not met in systems where applied voltages and electrolyte concentrations exceed certain limits. It is a well-known practical issue that is frequently faced in Electrochemistry. For instance, even in alkaline water electrolyzers for hydrogen generation, deviations from linearity are often experimentally observed due to the convection phenomena, while the applied voltages in such systems are only about 2 V or frequently slightly below (current densities are on the order of hundreds of milliamps). It is absolutely not clear why the Readers expect the situation with anodizing in, e.g., 10 wt % H_3_PO_4_ at 160 V, to be much different.

I think the necessary practical experience in electrochemistry could help the Readers to construct a new or completely revised mathematical model, this time reinforced by a well-developed scientific intuition. For example, polarization curves for similar systems containing metallic working electrodes immersed into a corrosive aqueous solution (e.g., recorded using the potentiodynamic polarization method—PDP) demonstrate that the linear (Ohmic) curve parts are usually located at a distance between 50 and 250 mV from the open circuit potential OCP (i.e., a steady-state potential that is established after the immersion of a metallic sample into solution without application of any external potential). The deviation from Ohm’s law is experimentally observed even before any considerable chemical modification of the metal surface due to RedOx reactions (dissolution, passivation, evolution of reactive gases, etc.), and is associated predominantly with a build-up of a concentration gradient at the electrode surface (so-called Stern deviations from the straight lines that lend themselves to Tafel extrapolation method). There is a reason why, for example, the linear polarization resistance (LPR) scans are performed with a small amplitude of 40–50 mV around OCP. An electrochemist who already has relevant practical experience with such metal-solution systems can often intuitively predict what behavior is realistic to expect.

## 4. Analyzing the “Overall Neutrality Condition”

In the Comment, the Readers state that “*MP didn’t discuss or in any way disproves the mathematical basis or model of our theory at any point of his writing, he doesn’t explain, why the results should become arbitrary*”. They also write their equation for a so-called “overall neutrality condition” that is aimed to show that “*Faraday’s laws are satisfied*”. At the Readers’ request, I analyze their equation in this section.

The equation is as follows:

(5) $$c_{red,H^{+}}=c_{Al,H^{+}}+\sum_{A}c_{A,H^{+}}$$

where:

$c_{red,H^{+}}$—“*the complete amount of reduced H^+^”* in *3H^+^ + 3e^−^ → 3/2H_2_* (Equation 3 in the Readers’ paper [4])

$c_{Al,H^{+}}$—“*the amount of H^+^ needed to compensate the charge of Al^3+^-ions*”, this charge is generated in *Al →Al^3+^ + 3e^−^* (Equation 4 in the Readers’ paper [4])

$\sum_{A}c_{A,H^{+}}$—“*the amount of H^+^ needed to compensate the charge of anions incorporated in the film.*”

The Readers also write that “*Anions have a negative charge which is compensated by associated Al^3+^-ions in the film.*”

For simplicity and clarity, let us consider the case with anodizing in H_2_SO_4_ solution and simply write anions as SO_4_^2−^. The foregoing quoted sentence then simply means that all anions incorporated into the PAA film exist there as sulphate salt Al_2_(SO_4_)_3_.

It is not clear why $c_{red,H^{+}}$ is called “*the complete amount of reduced H^+^*” because the water oxidation half-reaction 2H_2_O → 4H^+^ + 4e^−^ + O_2_ is not included into the right (anodic) part of their algebraic equation, but let us simply analyze what is on the table. We just need to keep in mind that $c_{red,H^{+}}$ is just a fraction of all reduced protons at the cathode that balances only oxidation of Al, but not water.

Let us now rewrite the $c_{red,H^{+}}=c_{Al,H^{+}}$ part of the Equation (5) using chemical notation, i.e., balance the charges using Equations 3 and 4 from the Readers’ work [4]:

Al + 3H^+^ → Al^3+^ + 3/2H_2_ (6)

Now let us add the process of incorporation of anions into the film where their charge is balanced by Al^3+^, as written by the Readers:

Al + 3/2H_2_SO_4_ → 1/2Al_2_(SO_4_)_3_ + 3/2H_2_ (7)

Here, SO_4_^2−^ in the left part are in the solution, and in the right part they are already incorporated into PAA. Now let us analyze the changes after adding the anions incorporation process to the RedOx equation from the electrochemical standpoint.

- Does sulphate participate in any RedOx process? The correct answer is “no” because none of the elements in anions change their oxidation states.
- Does sulphate send any electrons to an external circuit, so that they have to be replenished at the cathode by H^+^ reduction? The correct answer is also “no”, this follows from the answer to the previous question.

Thus, the problem is solved: the term of the equation $\sum_{A}c_{A,H^{+}}$ is superfluous, or, in mathematical terms:

$$\sum_{A}c_{A,H^{+}}=0$$

I think that the result of this analysis can be formulated in the best possible way just by repeating the conclusion from my review in *Nanomaterials* (p. 52): “*the actual mechanism of anionic impurity incorporation into PAA is very likely akin to ion exchange when electrolyte anions simply replace OH^−^ ions within PAA (see Figure 16). Thus, no excess charge is created within the solution, and therefore, no electrode processes need to be additionally involved*” [2].

## 5. What Does the Derivation of “*P*” (or “*p*”) Have to Do with Thermodynamics?

In this section, I would like to address the Readers’ constant reminders in the Comment that their estimates are products of a precise and exact science, whereas all my arguments are “*difficult to scientifically substantiate*” etc. In particular, two following fragments of the Comment have attracted my attention:

- *“In contrast to MP’s statement, the non-constant character of p is a direct consequence of its relation to entropy-production in our theory (see our paper p. 11919 conclusions)”*
- *“The derivation of the p-factor from a mathematically and physically correct framework shows how p is related to the entropy production of a system which owns a stationary state including a constant current, which results from non-stationary conditions. It was shown by us that the entropy production and p solely depend on migration and that diffusion and convection are negligible”*

I have gone through the chapter 2.5 called in their paper “Connection of local entropy production and electrolyte bulk properties” again. This is the chapter where the Readers demonstrate their derivation of *P*. Interestingly, I have not found any thermodynamic calculations, or any mentioning of thermodynamic parameters and functions such as entropy, at all. The chapter contains some typical equations for the coupling of electric field to fluid flow in nonequilibrium systems. The species transport equations are connected to the charge transport equations in a standard manner, by using ionic charges and the Faraday constant. This chapter is also preceded by some use of the Nernst–Planck equations, which are the basic conservation equations for species. Maybe I am missing something here, but where is the relation of *P* to entropy generation? Only in Conclusions?

The same applies to their removal of convection from the mass and charge transport mechanisms responsible for the hexagonal pattern formation and assigning the leading role to migration on the basis of entropy production. The well-known limitation of the thermodynamic method is that it uses parameters known from an experiment and does not allow any conclusions about materials structure or mechanisms of processes. This is what P.W. Atkins called in his Physical Chemistry book “the power and the poverty of thermodynamics”: “*The power is that we are able to relate one quantity, which might be difficult to measure directly, to other properties, which might be easily measured. The poverty is that the results are merely mathematical identities: they rely on mathematical manipulations which, being independent on the physics, give little information about the microscopic nature of matter*” [11].

The Readers’ paper in which they argue that “*a connection between the generation of entropy and the size of PAOX cells is evident*” creates a feeling that it is really little based on the power of thermodynamics [4]. On the contrary, the poverty of the method (in Atkins’ metaphoric definition) is plainly demonstrated.

## 6. The Readers’ Model: A Hypothesis, a Theory, or a Law That Must Pass the Test of Time?

The Readers wrote in their Comment: “*the porosity number p is a result of our derivation, the latter could reflect experimental reality since the validity of p was experimentally proven by MP itself in a joint paper together with the author Jörg J. Schneider* [4]*. Hence, the result of our theory is therefore an experimentally proven parameter, which confirms our arguments and ensures comparability to the experiment.*”

This conflicts with Logic and Methodology of Science (especially with the Confirmation Theory), which states that a hypothesis is formulated before obtaining evidential support, not after it.

Development of the electroconvective model was my independent and curiosity-driven “pet project” during PhD on a different topic in 2006–2010. I published a series of papers supporting the theory by experimental evidence in 2011–2020, and included my then-professor as a co-author into manuscripts, which is a common practice.

However, I have to say that all my experimental tests have been performed as part of the work on the electroconvective theory confirmation. For instance, the prediction of new electrolytes was tested in 2011 (formic acid) and 2013–2017 (tartronic acid), the theory proved to be consistent with new pulse current anodizing experiments in 2013, morphological features of nanopores resulting from electroconvective turbulence were microscopically observed in 2017 (and provided explanation for the pore branching mechanism), the universal dependency of the critical value of *P* on ionic concentrations was published in 2016, and, finally, submillimetre honeycomb structures resulting from electroconvective vortices expansion were presented to scientific community in 2020 (the paper has been selected by RSC Editors as a *2020 Hot PCCP article*) [5,6,7,12,13,14,15]. Moreover, the theory received additional support in experiments conducted by the Warsaw group (Stępniowski et al.) and Indian groups collaborating with the Erlangen group in Germany (Chelliah et al.) [16,17]. Thus, this theory proved out over the years.

I never performed any experiments to confirm or disconfirm the alternative hypothesis proposed by the Readers by evidence. Thus, my experimental testing results cannot be considered as empirical evidence for their model, especially those reported 3–9 years before its appearance.

Without going deep into analytic philosophy, an infinite number of alternative hypotheses that account for the same old body of evidence can be created. If the Readers want to increase the likelihood that their hypothesis is stronger than the alternative ones, they must design their own experimental testing program and provide new evidential support that will point out its advantages.

If their hypothesis has been specially formulated in such way that old evidence can be presented, then such evidence provides no support for their hypothesis.

Needless to say, the Readers’ claim that “*“Pashchanka theory” (in form of the factor p), is the conclusion from our theory and their assumptions*” is also absolutely unjustified from the chronological standpoint because I proposed the idea of a critical number “*P*” almost a decade earlier.

Remarkably, the Readers also call their model a “theory” several times in the Comment. In my opinion, it is too early to attribute such status to their model. A theory not only explains known facts, it should also be able to predict what should be observed if a theory is true. The Readers should demonstrate testability of their “theory” by providing predicted experimental observations compatible exclusively with their model. Until now, it has passed no test of predicting ability.

There are understudied systems that could help to test the Readers’ formula. For example, Ovechenko et al. have recently demonstrated the growth of disordered PAA structures in chemically pure water and alcohols [18]. I think, this could be a beautiful case study for the Readers’ model that could make it closer to the status of a theory. If their new formula (based on ionic strength) helps to deliberately change the anodizing conditions in deionized water or ethylene glycol in such way that the resulting PAA cells take a regular hexagonal shape, this could reinforce their work.

## 7. Concluding Remarks

The Readers have also tackled many other interesting topics in the Comment that undoubtedly deserve detailed discussion. However, I think that I have properly addressed the most important raised issues, and adding more topics may divert attention from the real core of the problem.
